# Liver-Directed Concurrent Chemoradiotherapy versus Sorafenib in Hepatocellular Carcinoma with Portal Vein Tumor Thrombosis

**DOI:** 10.3390/cancers14102396

**Published:** 2022-05-12

**Authors:** Jina Kim, Hwa Kyung Byun, Tae Hyung Kim, Sun Il Kim, Beom Kyung Kim, Seung Up Kim, Jun Yong Park, Do Young Kim, Jinsil Seong

**Affiliations:** 1 Department of Radiation Oncology, Yonsei Cancer Center, Yonsei University College of Medicine, Seoul 03722, Korea; jinabelle@yuhs.ac (J.K.); hkbyun05@yuhs.ac (H.K.B.); thkim@eulji.ac.kr (T.H.K.); 2 Department of Pathology, Yonsei University College of Medicine, Seoul 03722, Korea; alexkim94@yuhs.ac; 3 Division of Gastroenterology, Department of Internal Medicine, Yonsei University College of Medicine, Seoul 03722, Korea; beomkkim@yuhs.ac (B.K.K.); ksukorea@yuhs.ac (S.U.K.); drpjy@yuhs.ac (J.Y.P.); dyk1025@yuhs.ac (D.Y.K.)

**Keywords:** carcinoma, hepatocellular, thrombosis, chemoradiotherapy, sorafenib, prognosis

## Abstract

**Simple Summary:**

We investigated the efficacy of liver-directed concurrent chemoradiotherapy compared with sorafenib in hepatocellular carcinoma patients with portal vein tumor thrombosis. Patients in the sorafenib group presented higher incidences of unfavorable clinical features, and propensity score matching was performed to compensate for the differences between the two groups. We found that liver-directed concurrent chemoradiotherapy resulted in significantly improved survival compared to the sorafenib group. 3.6% and 13.8% of patients in the sorafenib and liver-directed concurrent chemoradiotherapy groups underwent surgical treatment after initial treatment, and those who received surgical treatment had significantly longer overall survival.

**Abstract:**

This study aimed to investigate the efficacy of liver-directed concurrent chemoradiotherapy (LD-CCRT) compared with sorafenib in patients with liver-confined locally advanced hepatocellular carcinoma (HCC) presenting portal vein tumor thrombosis (PVTT). This single institute retrospective cohort study included patients treated with sorafenib or LD-CCRT between 2005 and 2016. Patients with extrahepatic disease and those without PVTT were excluded, leaving 28 and 448 patients in the sorafenib and LD-CCRT groups, respectively. Propensity score matching was performed to balance the differences in clinical features between the two groups. At baseline, the sorafenib group presented higher incidences of unfavorable clinical features, including type III-IV PVTT (53.6% vs. 30.6%, *p* = 0.048) and bilateral disease extent (64.3% vs. 31.5%, *p* = 0.001), than the LD-CCRT group. A total of 27 patients from the sorafenib group and 52 patients from the LD-CCRT group were matched. At a median follow-up of 73 months, the median overall survival (OS) was 4.3 and 9.8 months in the sorafenib and LD-CCRT groups, respectively (*p* = 0.002). Patients with PVTT type II and higher benefited more from LD-CCRT in terms of OS. The Cox proportional hazard model showed that LD-CCRT was a significant prognostic factor for OS. One patient from the sorafenib group and seven patients from the LD-CCRT group underwent curative surgical treatment. Patients who underwent surgical treatment had significantly longer OS. In conclusion, LD-CCRT showed superior survival outcomes to sorafenib in HCC patients with PVTT. LD-CCRT needs further consideration for its substantial local tumor control that can enable curative surgical treatment in selected patients.

## 1. Introduction

Systemic therapy has long been recommended for the treatment of advanced hepatocellular carcinoma (HCC), mainly involving sorafenib and, more recently, atezolizumab plus bevacizumab [1,2]. In locally advanced HCC, however, liver-directed locoregional therapy has shown substantial efficacy. In recent phase 3 trials comparing transarterial radioembolization (TARE) and sorafenib, overall survival did not significantly differ between the two groups [3,4]. Considering TARE is an internal radiotherapy, these results might further be extended to liver-directed combined radiotherapy (RT), which needs more attention in view of its efficacy, as well as enabling curative resection although limited [5,6,7]. Liver-directed concurrent chemoradiotherapy (LD-CCRT), which combines local RT with concurrent hepatic arterial infusional chemotherapy (HAIC) of 5-fluorouracil followed by monthly HAIC [8,9], has shown efficacy in downstaging locally advanced HCC that was initially deemed unresectable, consequently improving the survival outcomes of patients [6,7]. More recently, it was found that liver-directed combined RT could convert tumors beyond the Milan criteria to within the Milan criteria in selected patients [5].

In particular, portal vein tumor thrombosis (PVTT) is a key dismal prognostic factor in HCC [10]. HCC with PVTT was previously considered unsuitable for treatments with curative intent, including locoregional therapies. However, accumulating evidence during the last two decades has shown favorable outcomes after RT [11,12,13]. RT alone has been reported to show an objective response rate of >50% in HCC with PVTT [14]. Moreover, transcatheter arterial chemoembolization (TACE) plus RT has been proven superior to both TACE alone and sorafenib in patients with locally advanced HCC [15,16,17]. Although such findings clearly suggest the efficacy of RT in HCC with PVTT, comparative studies with systemic therapy have been limited.

In this study, we aimed to investigate the treatment outcomes of LD-CCRT and sorafenib in patients with liver-confined locally advanced HCC with PVTT. To our knowledge, this is the first attempt to compare the treatment outcomes of LD-CCRT and sorafenib in locally advanced HCC.

## 2. Materials and Methods

### 2.1. Study Population

**Figure 1:**
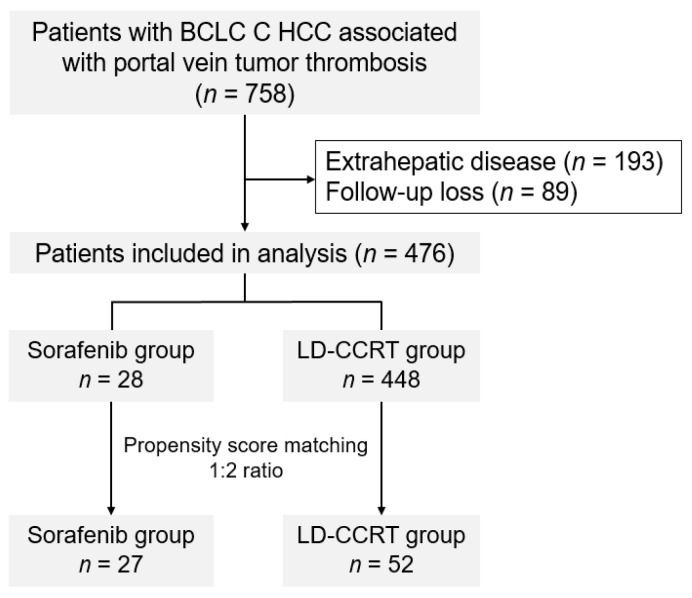
CONSORT (Consolidated Standards of Reporting Trials) diagram.

A list of consecutive patients who were diagnosed with primary HCC and treated with sorafenib or LD-CCRT between January 2005 and October 2016 was extracted and reviewed. The inclusion criteria were as follows: (a) radiologically or histologically diagnosed HCC, (b) disease confined to the liver, and (c) presence of PVTT. Patients with metastatic regional lymph nodes adjacent to the main hepatic mass were included in the study. Patients with extrahepatic disease, those without evidence of PVTT, and those who were lost to follow-up were excluded from the study. After all exclusions, the data of 28 patients in the sorafenib group and 448 patients in the LD-CCRT group were analyzed (Figure 1). This study was approved by the Institutional Review Board (approval no. 4-2020-0498) of Yonsei University College of Medicine, Seoul, Republic of Korea, and the requirement for informed consent was waived owing to the retrospective study design.

### 2.2. Treatment

Treatment was decided after a thorough discussion of the multidisciplinary team consisted of a medical oncologist, hepatologist, radiologist, radiation oncologist, hepatobiliary surgeon, and pathologist at our institution. In particular, patients’ general condition, liver function, and presence of RT-targetable lesions were considered.

For patients in the sorafenib group, 400 mg sorafenib twice a day was initially administered. The dose was modified if adverse reactions occurred, and treatment was continued until disease progression or patient intolerance. All patients in the LD-CCRT group received 3D conformal or intensity-modulated RT. All available diagnostic images, including CT, MRI, and PET-CT, were considered for RT planning. Planning CT was performed in all patients, and abdominal compressors were used to minimize respiratory movement in tolerable patients. Gross tumor volume was defined as the main hepatic tumor and the PVTT. Adjacent regional lymph nodes were included in the RT field if present and intrahepatic metastatic nodules were included in the RT field if located close to the main tumor. Before 2010, a generous margin in the craniocaudal direction was set to account for respiratory movements. Since 2010, 4D CT has been adopted at our institution, and tumor movements in all respiratory phases have been delineated for defining the internal target volume. The clinical target volume was defined as a margin around gross tumor volume to encompass microscopic disease extent. The planning target volume was defined as a 5-mm expansion in all directions from the clinical target volume. Concerning the RT dose, 45 Gy in 25 fractions was prescribed to the planning target volume for patients receiving RT with 3D conformal radiotherapy. With the introduction of intensity-modulated RT, a simultaneous integrated boost of 50–75 Gy in 25 fractions was prescribed to the internal target volume and 45–60 Gy in 25 fractions was prescribed to the planning target volume. LD-CCRT was performed with RT and concurrent HAIC, and 5-fluorouracil (500 mg/m^2^/day) was administered during the first and last weeks of the 5-week RT course. Additional HAIC after LD-CCRT involved the administration of 5-fluorouracil (500 mg/m^2^ on days 1–3) and cisplatin (60 mg/m^2^ on day 2) every 4 weeks starting at 1 month after completion of LD-CCRT which was continued until tumor progression or patient intolerance [5,8,18].

Patients underwent surgical treatment of either liver resection or liver transplantation when the multidisciplinary team decided that the tumor showed treatment response and no newly developed lesions were found upon follow-up evaluations after initial treatment. Specifically, liver resection was performed when tumors regressed to a resectable state, limited to either the right or left side of the liver, with no evidence of major vessel invasion, and when a substantial volume of future remnant liver was expected. Patients with focal PVTT were able to undergo a surgical procedure. Liver transplantation was performed according to the Milan criteria. However, in few cases, liver transplantation from a living donor was performed upon patient request since organ donation among family members occurs not infrequently in Korea. Nonetheless, major vessel invasion and the presence of extrahepatic disease were contraindications in such cases too.

### 2.3. Evaluation of Treatment Response and Statistical Analysis

Treatment response was evaluated through follow-up image studies performed every 1–3 months after completion of each treatment course. The longest diameters of tumors were measured and treatment response was judged based on the modified Response Evaluation Criteria in Solid Tumors (mRECIST) [19].

The baseline characteristics were compared between the two groups using the chi-square test, Fisher’s exact test, or Student’s *t*-test, as appropriate. The Kaplan–Meier method and log-rank test were used to analyze overall survival (OS), progression-free survival (PFS), locoregional recurrence-free survival, and distant metastasis-free survival. OS was defined from the date of treatment initiation to the date of death or the last follow-up. PFS was defined from the date of treatment initiation to the date of disease progression, relapse, initiation of a new unplanned anticancer therapy, disease-related death, or the last follow-up. Locoregional recurrence-free survival and distant metastasis-free survival were defined from the date of treatment initiation to the date of locoregional recurrence and distant metastasis, respectively.

We used propensity score matching (PSM) to overcome the differences in patient and tumor characteristics. PSM was performed to account for differences in the following factors: patient age, main tumor size, PVTT type (Cheng’s classification), and disease extent (unilateral or bilateral). Patients in the sorafenib and LD-CCRT groups were matched at a 1:2 ratio with a caliper width of 0.2 SD, resulting in 27 patients in the sorafenib group and 52 patients in the LD-CCRT group. Patients with identical scores were matched and nonmatched patients were eliminated.

Statistical significance was set at a *p*-value of <0.05. The Cox proportional hazard regression model was used for multivariable analysis. Hazard ratios and 95% confidence intervals were calculated to determine statistical significance. Statistical analyses were conducted using IBM SPSS Statistics for Windows (version 25.0; IBM Corp., Armonk, NY, USA).

## 3. Results

### 3.1. Patient and Treatment Characteristics

The patient and tumor characteristics are summarized in Table 1. Before PSM, the sorafenib group presented higher incidences of unfavorable tumor factors than the LD-CCRT group: PVTT type III or IV (53.6% vs. 30.6%, *p* = 0.048) and bilateral disease (64.3% vs. 31.5%, *p* = 0.001) were more frequently observed in patients treated with sorafenib than in those treated with LD-CCRT. In terms of patient age, tumor size, lymph node metastasis, Eastern Cooperative Oncology Group performance score, and Child–Turcotte–Pugh score, no significant differences were observed between the two groups.

PSM was performed to account for the differences between the two groups. Age at diagnosis, tumor size, PVTT type, and tumor extent were selected for PSM. After PSM at a 1:2 ratio, 27 and 52 matched patients remained in the sorafenib and LD-CCRT groups, respectively. The patient and tumor characteristics of the two groups before and after PSM are listed in Table 1. After PSM, all characteristics were well-balanced between the two groups. Most of the patients in the matched cohort had a viral infection (HBV or HCV) (94.9%, 75/79), and roughly two-thirds had bilateral disease (62.0%, 49/79). Of the 52 patients who received LD-CCRT, 18 (34.6%) patients received intensity-modulated RT while 34 (65.4%) patients received 3D-conformal radiotherapy.

### 3.2. Survival Analysis

**Figure 2:**
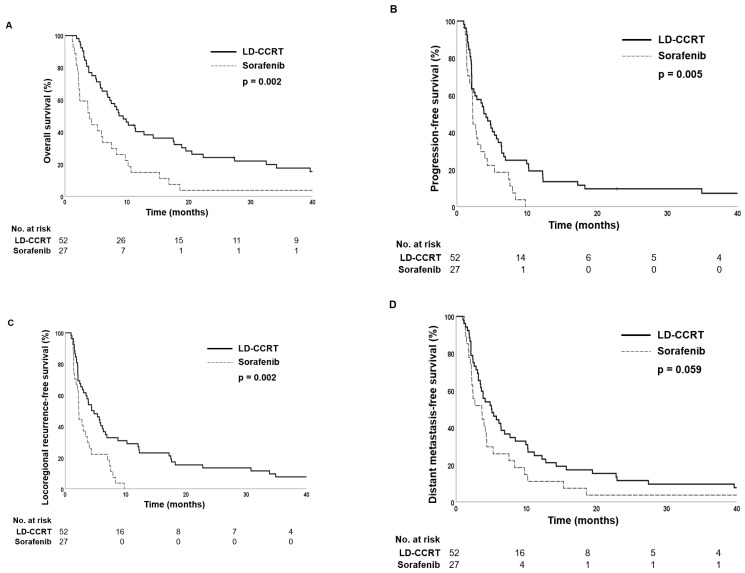
Kaplan–Meier estimates of (**A**) overall survival, (**B**) progression-free survival, (**C**) locoregional recurrence-free survival, and (**D**) distant metastasis-free survival in the propensity score-matched patients.

At a median follow-up of 73.1 months (range, 11.0–109.6 months), the median OS was 4.3 and 9.8 months in the sorafenib and LD-CCRT groups, respectively (*p* = 0.002) (Figure 2A). A survival benefit in the LD-CCRT group was also observed in terms of PFS and locoregional recurrence-free survival: the median PFS was 2.4 months in the sorafenib group and 4.6 months in the LD-CCRT group (*p* = 0.005), and the median locoregional recurrence-free survival was 2.2 months in the sorafenib group and 4.4 months in the LD-CCRT group (*p* = 0.002) (Figure 2B,C). In terms of distant metastasis-free survival, no significant difference was observed between the two groups: the median distant metastasis-free survival was 3.7 months in the sorafenib group and 5.0 months in the LD-CCRT group (*p* = 0.059) (Figure 2D).

Subgroup analysis showed that patients with PVTT type of II and higher benefited more from LD-CCRT in terms of OS, both in the propensity score unmatched and matched population (Figures S1 and S2). In patients with PVTT type I, 1-year OS rates were 75.5% for the LD-CCRT group and 50.0% for the sorafenib group in the total cohort, and 66.7% for the LD-CCRT group and 50.0% for the sorafenib group in the propensity score-matched population, both statistically insignificant (*p* = 0.110 and 0.364, respectively). In PVTT type II patients, 1-year OS rates were 58.6% for the LD-CCRT group and 18.2% for the sorafenib group in the total cohort (*p* < 0.001) and 45.8% for the LD-CCRT group and 18.2% for the sorafenib group in the propensity score-matched population (*p* = 0.012). In PVTT type III patients of the total cohort, 1-year OS rates were 40.9% and 14.3% for the LD-CCRT and sorafenib groups, respectively (*p* = 0.004). For PVTT type III patients, after propensity score matching, 1-year OS rates were 41.3% and 14.3% in the LD-CCRT and sorafenib groups, respectively (*p* = 0.027). A significant survival benefit was also observed for PVTT type IV patients in the total cohort; 1-year OS 54.5% vs. 0.0% (*p* = 0.038), but no analysis could be performed for the propensity score-matched patients since no patient received sorafenib treatment.

In univariate analysis, pretreatment alpha-fetoprotein value of ≤600 ng/mL, Child–Turcotte–Pugh class A, and LD-CCRT were prognostic factors associated with better OS. Pretreatment alpha-fetoprotein value of ≤600 ng/mL, and LD-CCRT remained as significant factors in the multivariate analysis (*p* = 0.005, and 0.001, respectively) (Table 2).

### 3.3. Treatment Response

**Figure 3:**
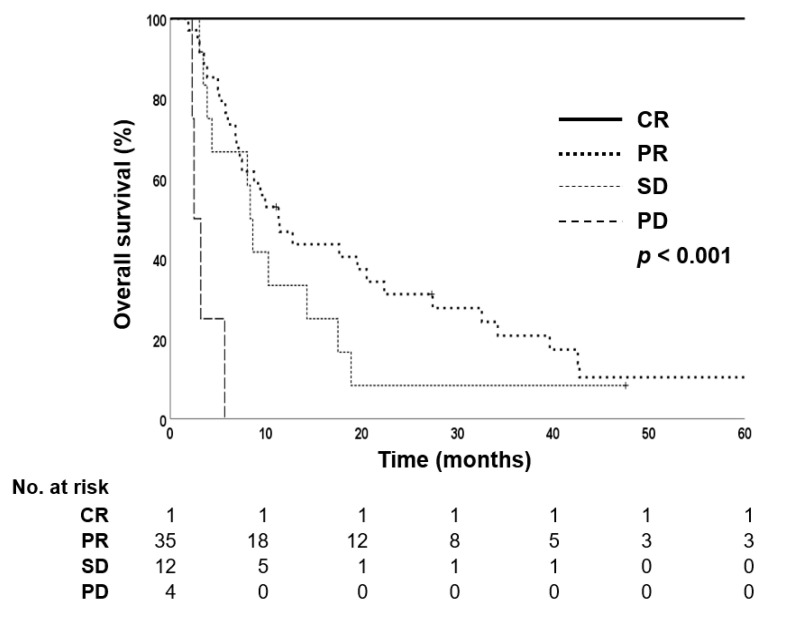
Kaplan–Meier estimates of overall survival in liver-directed concurrent chemoradiotherapy-treated group according to treatment response.

Treatment response was evaluated according to mRECIST criteria. Of the 52 patients who received LD-CCRT, 35 (67.3%) showed partial response or complete response in the follow-up imaging evaluation. In contrast, only two patients (7.4%) in the sorafenib group showed treatment response. In the LD-CCRT group, the 1-year OS was significantly higher in patients who showed treatment response than in those who did not respond to treatment: the 1-year OS rates were 100%, 46.7%, 33.3%, and 0.0% in patients with complete response, partial response, stable disease, and progressive disease, respectively (*p* < 0.001) (Figure 3).

**Figure 4:**
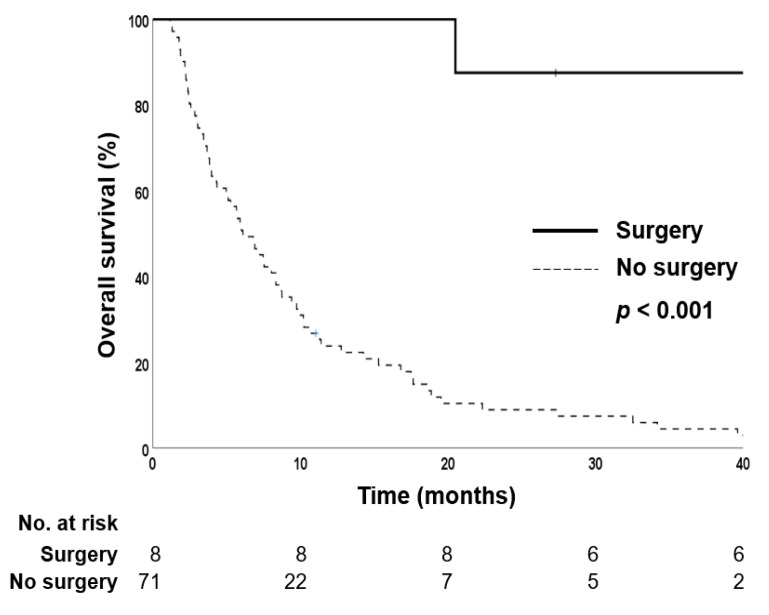
Kaplan–Meier estimates of overall survival in the propensity score-matched cohort according to surgical treatment.

**Figure 5:**
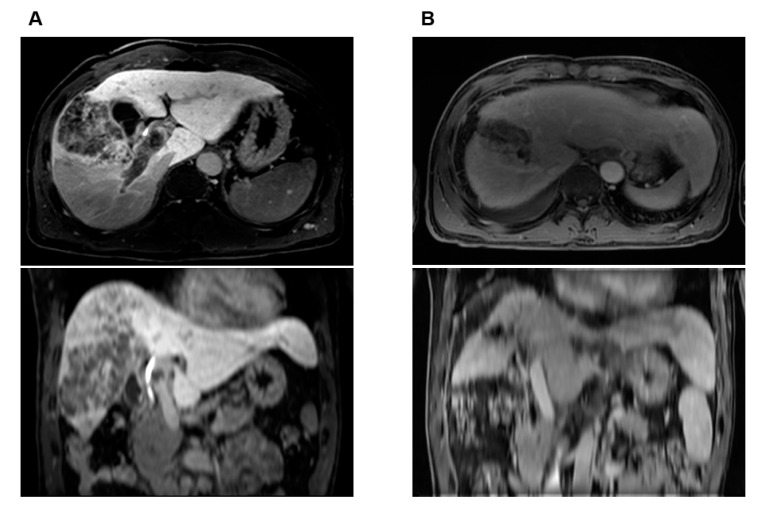
Case illustration of a 51-year-old male patient who received liver-directed concurrent chemoradiotherapy (LD-CCRT) followed by right lobectomy of the liver. Initial status was a 10.5 cm tumor involving both hepatic lobes associated with extensive tumor thrombosis involving right and main portal veins as well as elevated tumor markers. After LD-CCRT, significant tumor response was obtained with decreased tumor markers. Right lobectomy of liver was performed 4.5 months after the conclusion of LD-CCRT, and pathology report showed total necrotic tumor with no evidence of tumor thrombosis at portal vein. (**A**) Pretreatment axial and coronal MRI images showing locally advanced HCC with PVTT in right and main portal veins, (**B**) axial and coronal MRI images showing successful downstaging of tumor with reduced extent of PVTT 3 months after LD-CCRT. A dramatic decrease in tumor markers was also noted: alpha-fetoprotein and protein induced by vitamin K absence-II values of 63,430.93 ng/mL and 131 mAU/mL initially dropped to 4.97 ng/mL and 16 mAU/mL one month after LD-CCRT, respectively. The patient is alive without evidence of tumor recurrence at 101.5 months.

Out of the 28 patients in the unmatched sorafenib group, one (3.6%) patient received liver transplantation after sorafenib treatment. Of the 448 patients in the unmatched LD-CCRT group, 62 (13.8%) patients received surgical treatment—46 liver resections and 16 liver transplantations—after initial treatment. Among the propensity score-matched patients, one (3.7%) patient in the sorafenib group and seven patients (13.5%) in the LD-CCRT group underwent surgical treatment (liver resection or liver transplantation). Patients who underwent surgical treatment had significantly longer OS than those who did not undergo surgery (Figure 4). The patient from the sorafenib group underwent liver transplantation because of progressive disease and showed 5% tumor necrosis on surgical pathology. Of the seven patients who were treated with LD-CCRT followed by surgery, four patients showed 100% tumor necrosis and two patients showed 95% tumor necrosis on surgical pathology (Table 3). One case (case no. 5) of successful LD-CCRT followed by right lobectomy of the liver is shown in Figure 5.

### 3.4. Treatment-Related Toxicity

Treatment-related toxicity was evaluated by reviewing medical records and lab tests, and graded according to Common Terminology Criteria for Adverse Events version 4.0. Of the 27 patients in the propensity score-matched sorafenib group, three (11.1%) patients complained of grade 2 nausea, three (11.1%) had diarrhea (one grade 1 and two grade 2), four (14.8%) patients had poor oral intake (two grade 1 and two grade 2), two (7.4%) patients had grade 1 skin rash, and one (3.7%) patient had grade 2 hand-foot syndrome. Of the 52 patients who received LD-CCRT in the propensity score-matched cohort, one (1.9%) patient had grade 1 leukopenia, four (7.7%) had nausea (three grade 1 and one grade 3), three (5.8%) had grade 1 epigastric pain, two (3.8%) had grade 1 gastric ulcer, one (1.9%) had grade 3 stomach perforation, five (9.6%) had liver enzyme elevation, and one (1.9%) had grade 1 esophageal mucositis. The patient with stomach perforation underwent elective surgery. No grade 4 or 5 toxicity was observed in both groups.

## 4. Discussion

The present study investigated the oncologic outcomes of patients treated with LD-CCRT or sorafenib. The results demonstrated that LD-CCRT significantly improved the OS, PFS, and locoregional recurrence-free survival of HCC patients presenting with PVTT, relative to sorafenib. In this study, the median OS was 9.8 months after LD-CCRT and 4.3 months after sorafenib therapy. Substantial local tumor control eventually resulted in higher rates of curative surgery in the LD-CCRT group, in turn leading to longer survival.

In advanced HCC, the treatment aim is limited to palliation, as shown in a previous study on the long-term use of sorafenib [20]. The recent success of atezolizumab plus bevacizumab has offered a new chance of cure [2]. However, advanced HCC is subdivided into two categories: disease extending beyond the liver and disease confined to the liver. While systemic therapy is considered a standard therapy, liver-directed combination therapy can induce substantial local tumor control, which may open a chance for curative surgery. Our group has long been practicing liver-directed combined RT, in the form of either TACE plus RT or CCRT. The attractiveness of this approach seems to lie in effective local control, which will further allow conversion to curative surgery in selected patients. A pilot study from our institution reported a substantial response rate of 45% after RT concurrently administered with localized intra-arterial chemotherapy, with significantly prolonged survival in responders [8]. The addition of RT improved the survival of patients with locally advanced HCC treated with TACE compared with that of those treated with TACE alone [15,16]. A retrospective PSM analysis comparing TACE plus RT and sorafenib for advanced HCC also showed significant survival gain in the TACE plus RT group [17].

In particular, locally advanced HCC associated with PVTT represents the worst subgroup of liver-confined HCC. While sorafenib has been the standard of care for this subset of patients in the past, more aggressive treatment approaches are being taken in line with the view that PVTT is a complicated clinical condition in terms of its biological aggressiveness and patients’ general condition [21]. Intensive tumor control with liver-directed combined RT has been attempted in this subgroup of locally advanced HCC, with an increasing body of evidence supporting the benefit of RT. Yu et al. reported an objective response rate of 53.8% in HCC patients with PVTT after RT, with significantly prolonged survival in responders [14]. Zeng et al. evaluated the role of RT in HCC patients with PVTT and/or an inferior vena cava tumor thrombosis and reported an objective response rate of 45.5% after RT [22]. In another study comparing the treatment outcomes of neoadjuvant RT followed by surgery and upfront surgery in HCC patients with PVTT, the neoadjuvant RT group showed significantly improved OS and disease-free survival rates [10]. Such advances in treatment outcomes are noteworthy considering that HCC with PVTT was previously considered a dismal disease with no curative treatment.

Aikata et al. recently reported a high response rate after HAIC combined with RT—13.7% overall response rate in the main tumor and 51.0% overall response rate in the PVTT of the main trunk—with a higher median OS of responders [23]. The median OS and PFS were 12.1 and 4.2 months, respectively. This is an astonishing outcome considering that they only addressed HCC patients with tumor thrombosis of the main trunk or bilobar of the portal vein. The results from our data are comparable to the findings of the Japanese population. While the median OS and PFS of the LD-CCRT group in the propensity score-matched population is 9.8 and 4.6 months, respectively, the survival time escalates to 14.6 and 8.7 months before propensity score matching. Therefore, the survival outcomes of LD-CCRT should be interpreted with caution, so that the poor prognosis of the sorafenib group does not cast a shadow on the LD-CCRT group. In addition, the results of our data are noteworthy considering the fact that our data also included patients with heavy pre-treatment history, while Aikata et al. only reported survival outcomes of patients with no history of systemic therapy.

According to our data, patients with PVTT extending beyond the segmental branches of the portal vein showed greater survival gain through LD-CCRT both in the total cohort and the propensity score-matched population. A meta-analysis comparing the treatment efficacy of HAIC versus sorafenib also supports this finding [24]. In the meta-analysis, the advantages of HAIC over sorafenib were more obvious in HCC patients with types III-IV PVTT compared to patients with types II-IV PVTT. No clear explanation for such difference yet exists, and whether aggressive local treatment including LD-CCRT and HAIC is more suitable than sorafenib alone for the treatment of HCC with higher degrees of PVTT is a grey area and should be verified in the future.

While lower Child–Turcotte–Pugh class, lower pretreatment alpha-fetoprotein level, and LD-CCRT were significant factors predicting improved overall survival in univariate analysis, lower pretreatment alpha-fetoprotein level and LD-CCRT remained significant in multivariate analysis. A lower Child–Turcotte–Pugh score indicating better liver function and lower tumor marker level are well-known predictors of improved survival in HCC. Byun et al. demonstrated that lower Child–Turcotte–Pugh class and alpha-fetoprotein level predicted improved survival outcomes [18]. Kim et al. also showed that lower Child–Turcotte–Pugh class and alpha-fetoprotein level were significant predictors of prolonged survival [25]. Our finding has clinical implications in that while Child–Turcotte–Pugh class and alpha-fetoprotein level are patient or tumor-related factors that physicians cannot change, LD-CCRT is a factor we can modulate.

In this study, one patient from the sorafenib group and seven patients from the LD-CCRT group underwent surgical treatment with either liver resection or transplantation. Patients who underwent surgery had significantly longer survival than their counterparts. Notably, six of the seven patients who underwent surgery after LD-CCRT showed ≥ 95% tumor necrosis on pathology, whereas one patient who underwent surgery after sorafenib showed only 5% tumor necrosis. Our results are consistent with those of other studies. Lee et al. reported a 5-year OS rate of 49.6% in patients who underwent curative surgery, which was significantly higher than that of patients who did not undergo surgical treatment [6]. Kim et al. also reported excellent survival outcomes in patients who underwent surgical treatment after achieving tumor downstaging with LD-CCRT [26]. Han et al. analyzed the outcomes of living-donor liver transplantation for HCC with PVTT after LD-CCRT and reported a 1-year disease-free survival rate of 87.5% [27]. A recent study from our institution analyzed the treatment outcomes of liver-directed combined RT as a bridge to surgical treatment, and reported median OS in the no surgery, surgical resection, and liver transplantation groups of 12.7, 166.0, and 62.5 months, respectively [5]. Patients with younger age, single tumor, lower pretreatment Child–Turcotte–Pugh score, lower pretreatment tumor marker levels (alpha-fetoprotein, protein induced by vitamin K absence-II), and no previous treatment history were more likely to undergo surgical treatment after liver-directed RT. A meta-analysis by Zhang et al. concluded that patients without PVTT in the main trunk of the portal vein might gain more survival benefits through surgical approaches [28]. Therefore, careful selection of patients with the aforementioned favorable tumor characteristics might increase the chance of curative surgery after LD-CCRT.

The limitation of this study lies in its retrospective nature and limited sample size. The possibility of selection bias is nonnegligible although PSM analysis was performed to overcome the imbalance between the two treatment groups. In Korea, patients were able to get reimbursed for Sorafenib by the national health insurance system starting in 2011. This partly explains the limited number of patients in the Sorafenib group. Moreover, liver-directed RT has been widely performed for unresectable HCCs at our institution, thus limiting the number of patients in the sorafenib group. In addition, although new systemic agents, including lenvatinib or atezolizumab plus bevacizumab, have shown superior results in unresectable HCC [2,29,30], they were not considered in this study. Many questions remain unanswered, including the optimal RT dose and fractionation for locally advanced HCC. Further, whether LD-CCRT is compatible with other locoregional treatments (e.g., TACE and radiofrequency ablation) and with systemic treatments (e.g., sorafenib) needs to be further clarified. A phase II study in locally advanced HCC patients found that sequential sorafenib after LD-CCRT was effective in tumor control, with objective response rates after LD-CCRT and during sorafenib maintenance of 44.7% and 53.2%, respectively [26]. Future prospective multicenter studies with larger patient cohorts are necessary to further improve the survival outcomes through combination with systemic therapies.

## 5. Conclusions

In conclusion, LD-CCRT showed superior survival outcomes to sorafenib in locally advanced HCC patients with PVTT. LD-CCRT needs further consideration for its substantial local tumor control that can enable curative surgical treatment in selected patients. Further multi-institutional studies with larger patient cohorts are necessary to better assess the feasibility and efficacy of this novel treatment.

## Figures and Tables

**Table 1:** Baseline characteristics of the sorafenib- and liver-directed concurrent chemoradiotherapy-treated groups before and after PSM.

Characteristics		Before PSM					After PSM				
		Sorafenib		LD-CCRT		*p* Value	Sorafenib		LD-CCRT		*p* Value
		Median/No	Range/%	Median/No	Range/%		Median/No	Range/%	Median/No	Range/%	
Age		52.8	30.1–84.0	55.0	27.0–84.0	0.348	52.2	30.1–84.0	50.5	36.0–74.0	0.842
Tumor size		10.5	2.0–23.2	10.0	0.4–22.0	0.866	10.2	2.0–23.2	10.1	1.9–19.9	0.784
Gender	Male	23	82.1	395	88.2	0.367	22	81.5	49	94.2	0.114
	Female	5	17.9	53	11.8		5	18.5	3	5.8	
Viral factors	HBV	4	14.3	42	9.4	0.235	3	11.1	4	7.7	0.339
	HCV	24	85.7	371	82.8		24	88.9	44	84.6	
	None	0	0.0	35	7.8		0	0.0	4	7.7	
PVTT type (Cheng’s classification)	I	2	7.1	94	21.0	0.048	2	7.4	3	5.8	0.956
	II	11	39.3	217	48.4		11	40.7	24	46.2	
	III	14	50.0	126	28.1		14	51.9	24	46.2	
	IV	1	3.6	11	2.5		0	0.0	1	1.9	
LN metastasis	No	25	89.3	382	85.3	0.783	24	88.9	43	82.7	0.531
	Yes	3	10.7	66	147		3	11.1	9	17.3	
Disease extent	Unilateral	10	35.7	307	68.5	0.001	10	37.0	20	38.5	1.000
	Bilateral	18	64.3	141	31.5		17	63.0	32	61.5	
Pretreatment AFP	≤600 ng/mL	12	42.9	214	47.8	0.614	12	44.4	25	48.1	0.759
	>600 ng/mL	16	57.1	234	52.2		15	55.6	27	51.9	
ECOG Performance Status	0	7	25.0	122	27.2	0.475	7	25.9	14	26.9	0.482
	1	18	64.3	301	67.2		17	63.0	36	69.2	
	2	3	10.7	25	5.6		3	11.1	2	3.9	
Child-Turcotte-Pugh Score	5	11	39.3	165	36.8	0.189	11	40.7	18	34.6	0.447
	6	9	32.1	167	37.3		9	33.3	16	30.8	
	7	3	10.7	77	17.2		2	7.4	11	21.2	
	8	3	10.7	33	7.4		3	11.1	6	11.5	
	9	2	7.1	6	1.3		2	7.4	1	1.9	
Pretreatment history	No	27	96.4	357	79.7	0.030	26	96.3	46	88.5	0.245
	Yes	1	3.6	91	20.3		1	3.7	6	11.5	

Abbreviations: PSM, propensity score matching; LD-CCRT, liver-directed concurrent chemoradiotherapy; PVTT, portal vein tumorthrombosis; LN, lymph node; AFP, alpha-fetoprotein; ECOG, the Eastern Cooperative Oncology Group.

**Table 2:** Prognostic factors for overall survival in the propensity score-matched population.

Characteristics		*n*	Univariate		Multivariate	
			1 Year Survival	*p*	HR (95% CI)	*p*
Age	≤50	37	29.3	0.847		
	>50	42	33.3			
Viral factors	HBV	68	35.2	0.943		
	HCV	4	50.0			
	None	7	28.6			
Diabetes mellitus	No	62	32.2	0.055		
	Yes	17	47.1			
Tumor size	≤10 cm	39	38.3	0.204		
	>10 cm	40	25.0			
Venous invasion	No	60	40.0	0.273		
	Yes	19	21.1			
Intrahepatic metastasis	No	23	43.5	0.175		
	Yes	56	32.0			
PVTT type (Cheng’s classification)	I	5	60.0	0.639		
	II	35	31.4			
	III	38	28.5			
	IV	1	0.0			
LN metastasis	No	67	32.7	0.230		
	Yes	12	25.0			
Disease extent	Unilateral	30	33.3	0.581		
	Bilateral	49	30.6			
Pretreatment AFP	≤600 ng/mL	37	48.6	0.001	2.063 (1.243–3.425)	0.005
	>600 ng/mL	42	23.8			
ECOG Performance status	0	21	33.3	0.304		
	1	53	32.0			
	2	5	20.0			
Child-Turcotte-Pugh Class	A	54	36.9	0.021	1.446 (0.859–2.433)	0.165
	B	25	20.0			
Pretreatment history	No	72	30.5	0.336		
	Yes	7	71.4			
Treatment modality	Sorafenib	27	14.8	0.001	0.435 (0.265–0.714)	0.001
	LD-CCRT	52	40.2			

Abbreviations: HR, hazard ratio; CI, confidence interval; HBV, hepatitis B virus; HCV, hepatitis C virus; NBNC, non-B, non-C hepatitis; PVTT, portal vein tumorthrombosis; LN, lymph node; AFP, alpha-fetoprotein; ECOG, the Eastern Cooperative Oncology Group; LD-CCRT, liver directed-concurrent chemoradiotherapy.

**Table 3:** Patient and tumor characteristics in those who underwent surgical treatment.

No.	Age/Sex	Initial Tumor Size, cm	PVTT Type	Disease Extent	Initial Tx	Tx Response	Interval from Tx Initiation to Op (Months)	Op	Op Pathology	OS (Months)
1	58/M	7.8	III	Unilateral	Sorafenib	PD	44.2	Liver transplantation	Multiple viable HCCs, largest 2.5 × 1.0 cm, with 5% necrosis	73.1
2	51/M	15.7	II	Bilateral	LD-CCRT	PR	17.1	Lt. hepatectomy	Completely necrotic tumor	76.2
3	45/M	12	II	Bilateral	LD-CCRT	PR	6.3	Liver transplantation	Multiple viable HCCs, largest 2.8 × 2.8 cm, with 95% necrosis	79.0
4	51/M	8.6	I	Bilateral	LD-CCRT	PR	10.4	Lt. lobectomy	5 × 4 cm HCC, with 95% tumor necrosis	109.6
5	51/M	10.5	III	Bilateral	LD-CCRT	CR	5.6	Rt. lobectomy	Completely necrotic tumor	101.5
6	54/M	6.5	I	Bilateral	LD-CCRT	PR	18.2	Lt. lobectomy	2.5 × 2.5 × 1.8 cm HCC, with 50% tumor necrosis	20.5
7	50/M	5.2	III	Unilateral	LD-CCRT	PR	17.2	Liver transplantation	1.0 × 0.7 cm HCC, and three completely necrotic lesions	27.3
8	56/M	8	III	Bilateral	LD-CCRT	SD	46.9	Liver transplantation	Three completely necrotic lesions	50.2

Abbreviations: PVTT, portal vein tumorthrombosis; Tx, treatment; op, operation; OS, overall survival; LD-CCRT, liver-directed concurrent chemoradiotherapy; CR, complete response; PR, partial response; PD, progressive disease; SD, stable disease; Lt., left; Rt., right; M, male; HCC, hepatocellular carcinoma.

## Data Availability

Not applicable.

## Supplementary Materials

The following supporting information can be downloaded at: https://www.mdpi.com/article/10.3390/cancers14102396/s1, Figure S1: Subgroup analysis of the propensity score unmatched population. Kaplan-Meier estimates of overall survival in the (A) PVTT type I, (B) PVTT type II, (C) PVTT type III, and (D) PVTT type IV subgroup; Figure S2: Subgroup analysis of the propensity score matched population. Kaplan-Meier estimates of overall survival in the (A) PVTT type I, (B) PVTT type II, and (C) PVTT type III subgroup.
