# Supplementary material for: Liver-Directed Concurrent Chemoradiotherapy versus Sorafenib in Hepatocellular Carcinoma with Portal Vein Tumor Thrombosis
Source: Cancers (Basel). 2022 May 12;14(10):2396. doi: 10.3390/cancers14102396 (PMC9139919; doi:10.3390/cancers14102396)
Supplement: Supplementary file 1 [file cancers-14-02396-s001.zip › cancers-1701142-supplementary.pptx]

## Slide 1
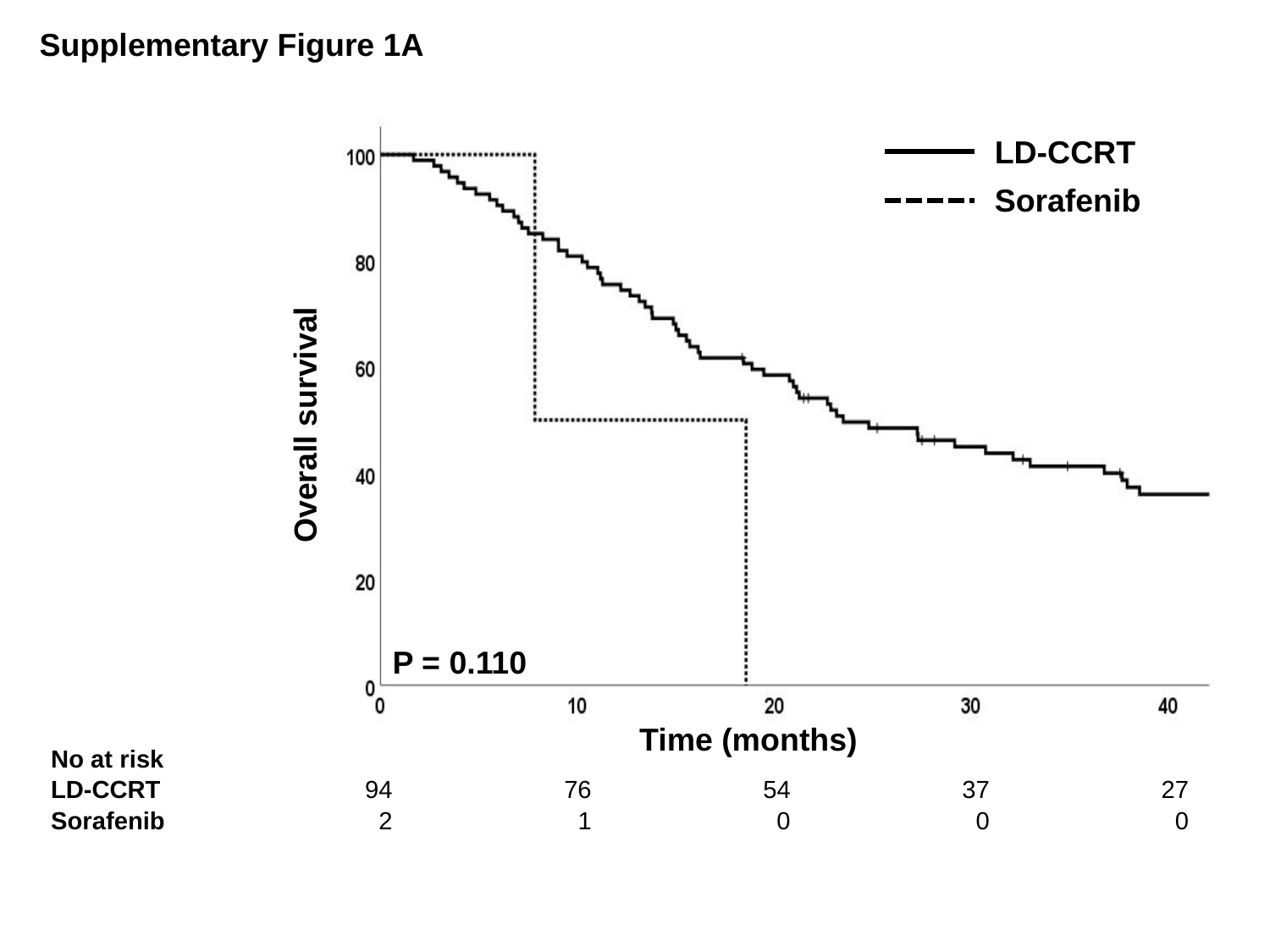

Supplementary Figure 1A
LD-CCRT
Sorafenib
Overall survival
P = 0.110
Time (months)
| No at risk | | | | | |
| --- | --- | --- | --- | --- | --- |
| LD-CCRT | 94 | 76 | 54 | 37 | 27 |
| Sorafenib | 2 | 1 | 0 | 0 | 0 |

## Slide 2
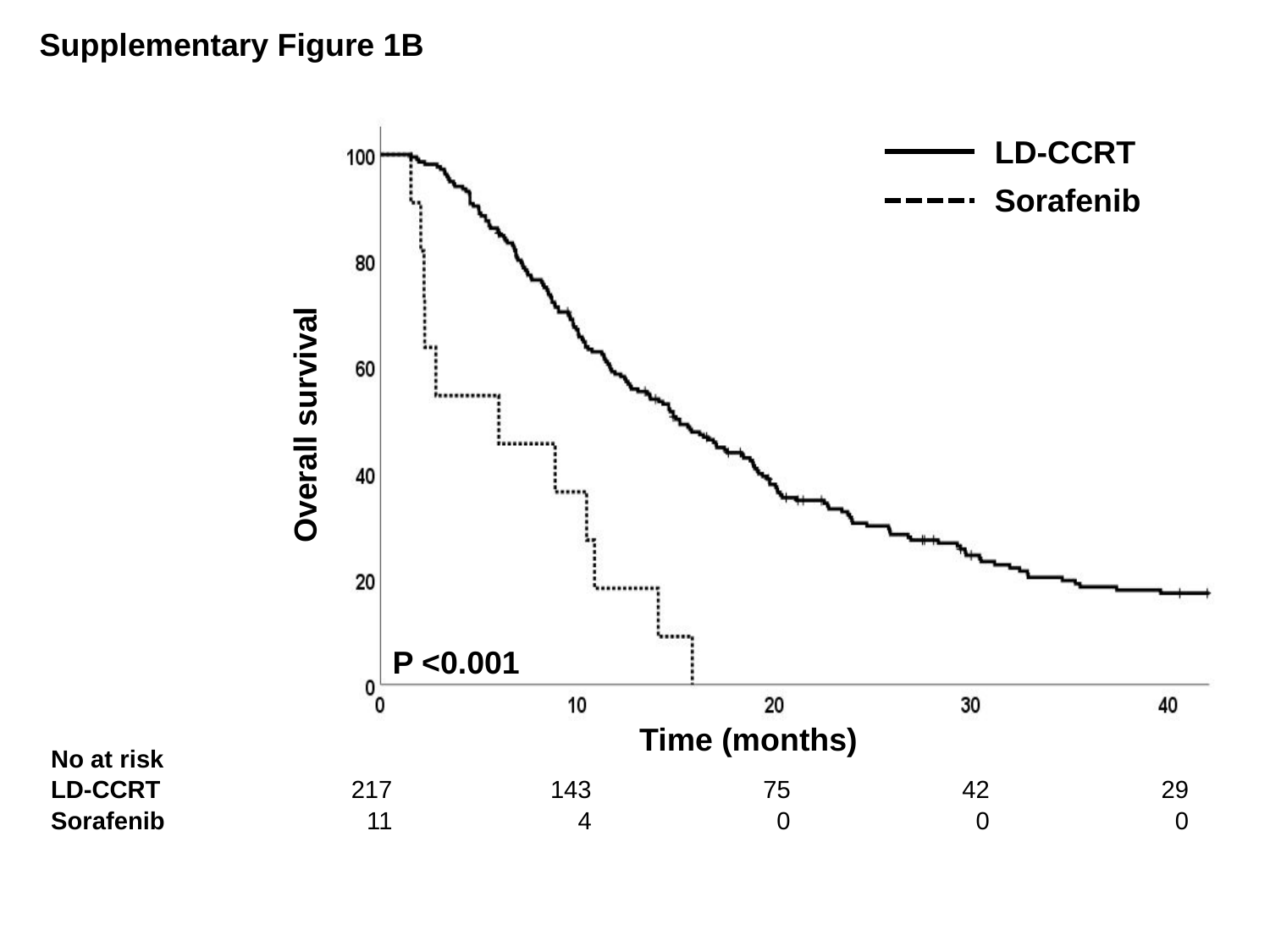

Supplementary Figure 1B
LD-CCRT
Sorafenib
Overall survival
P <0.001
Time (months)
| No at risk | | | | | |
| --- | --- | --- | --- | --- | --- |
| LD-CCRT | 217 | 143 | 75 | 42 | 29 |
| Sorafenib | 11 | 4 | 0 | 0 | 0 |

## Slide 3
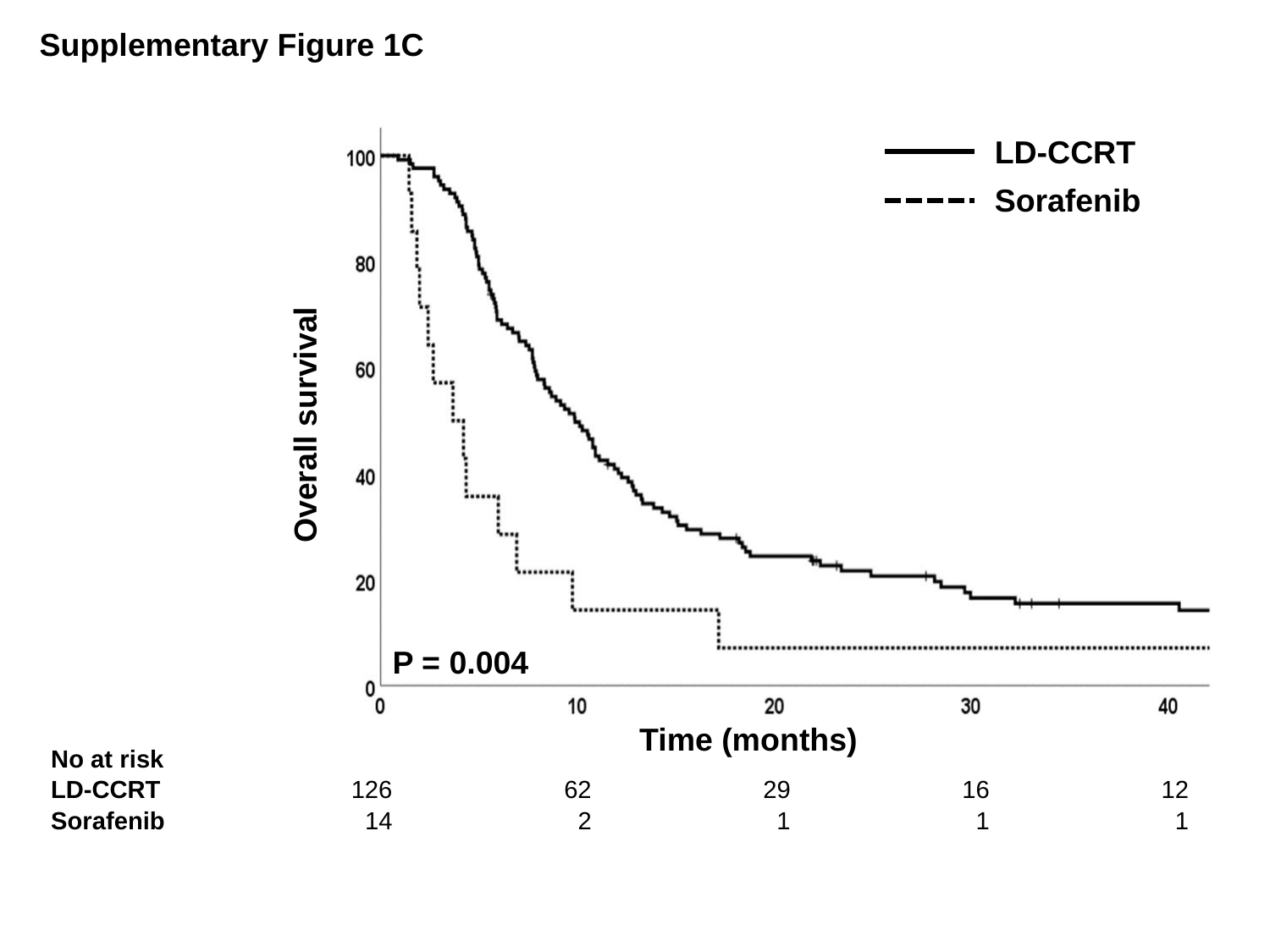

Supplementary Figure 1C
LD-CCRT
Sorafenib
Overall survival
P = 0.004
Time (months)
| No at risk | | | | | |
| --- | --- | --- | --- | --- | --- |
| LD-CCRT | 126 | 62 | 29 | 16 | 12 |
| Sorafenib | 14 | 2 | 1 | 1 | 1 |

## Slide 4
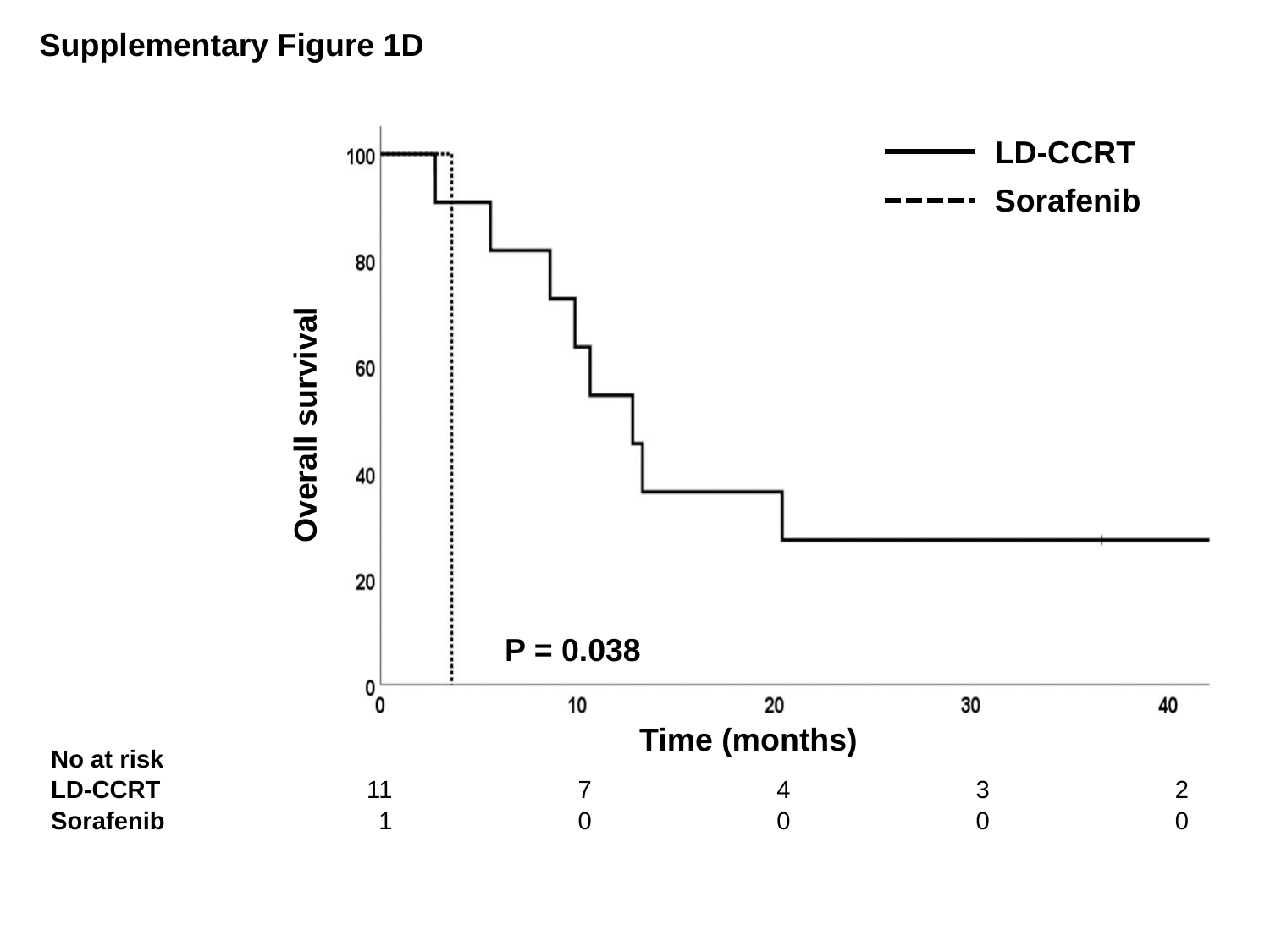

Supplementary Figure 1D
LD-CCRT
Sorafenib
Overall survival
P = 0.038
Time (months)
| No at risk | | | | | |
| --- | --- | --- | --- | --- | --- |
| LD-CCRT | 11 | 7 | 4 | 3 | 2 |
| Sorafenib | 1 | 0 | 0 | 0 | 0 |

## Slide 5
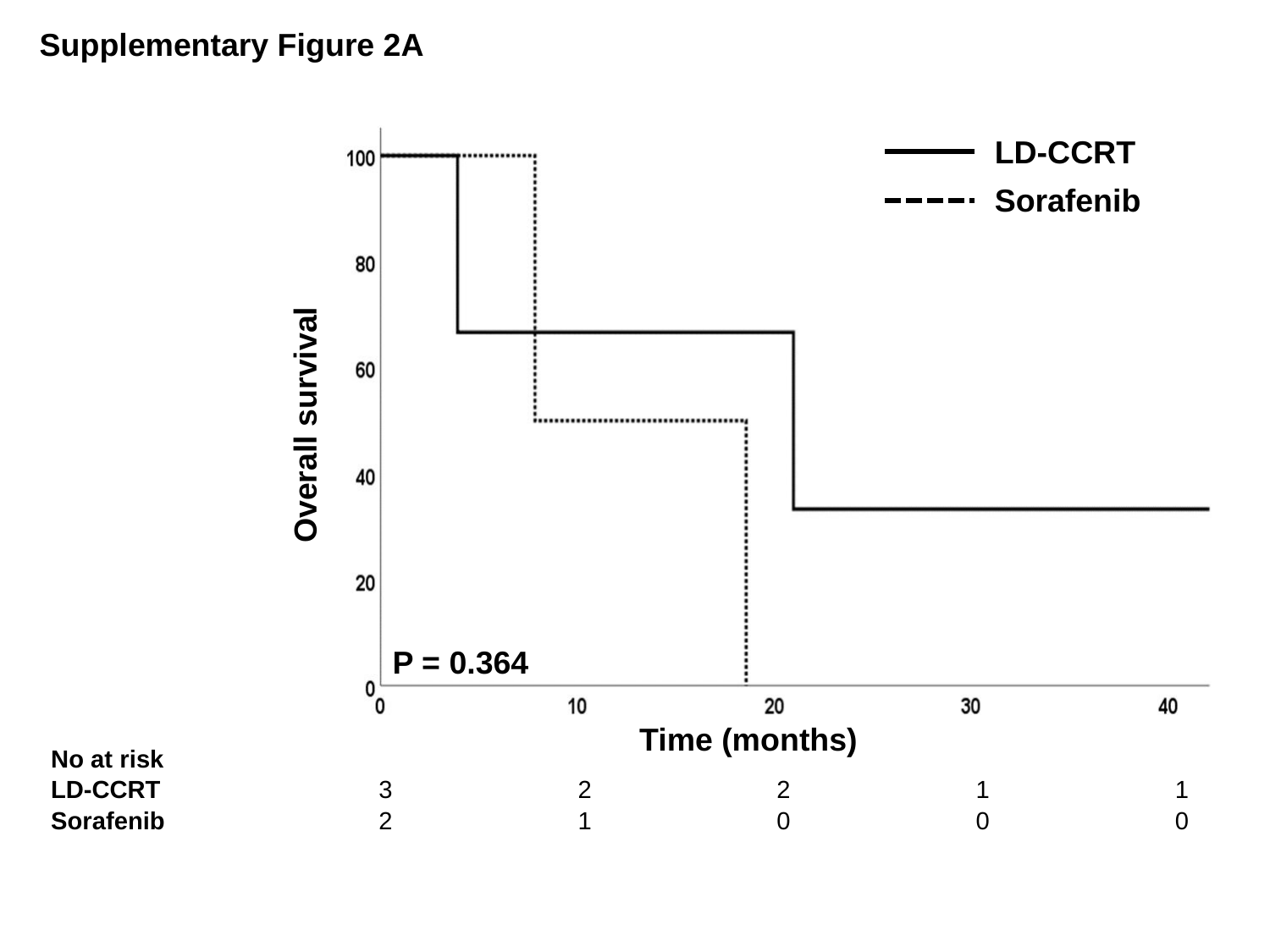

Supplementary Figure 2A
LD-CCRT
Sorafenib
Overall survival
P = 0.364
Time (months)
| No at risk | | | | | |
| --- | --- | --- | --- | --- | --- |
| LD-CCRT | 3 | 2 | 2 | 1 | 1 |
| Sorafenib | 2 | 1 | 0 | 0 | 0 |

## Slide 6
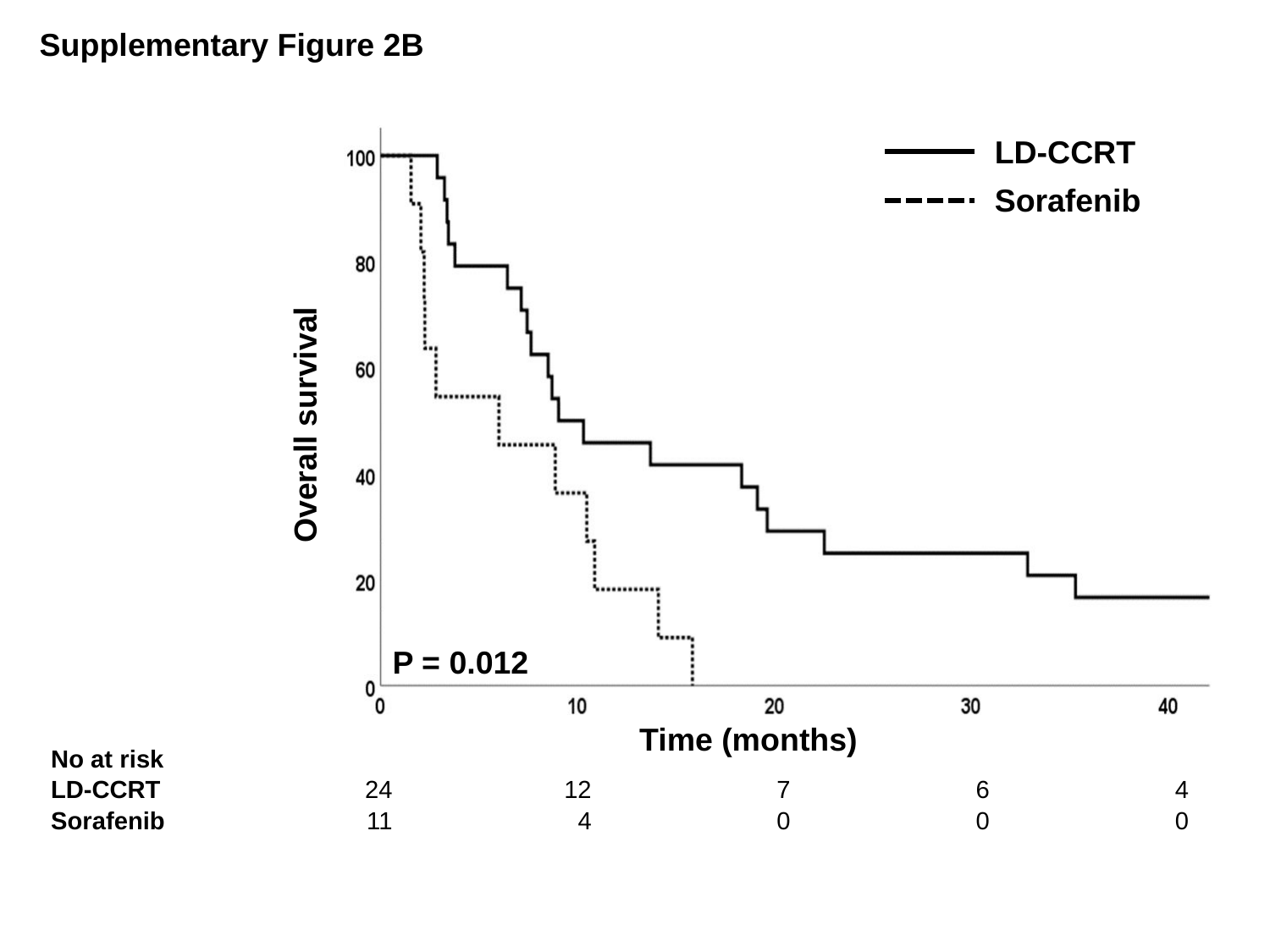

Supplementary Figure 2B
LD-CCRT
Sorafenib
Overall survival
P = 0.012
Time (months)
| No at risk | | | | | |
| --- | --- | --- | --- | --- | --- |
| LD-CCRT | 24 | 12 | 7 | 6 | 4 |
| Sorafenib | 11 | 4 | 0 | 0 | 0 |

## Slide 7
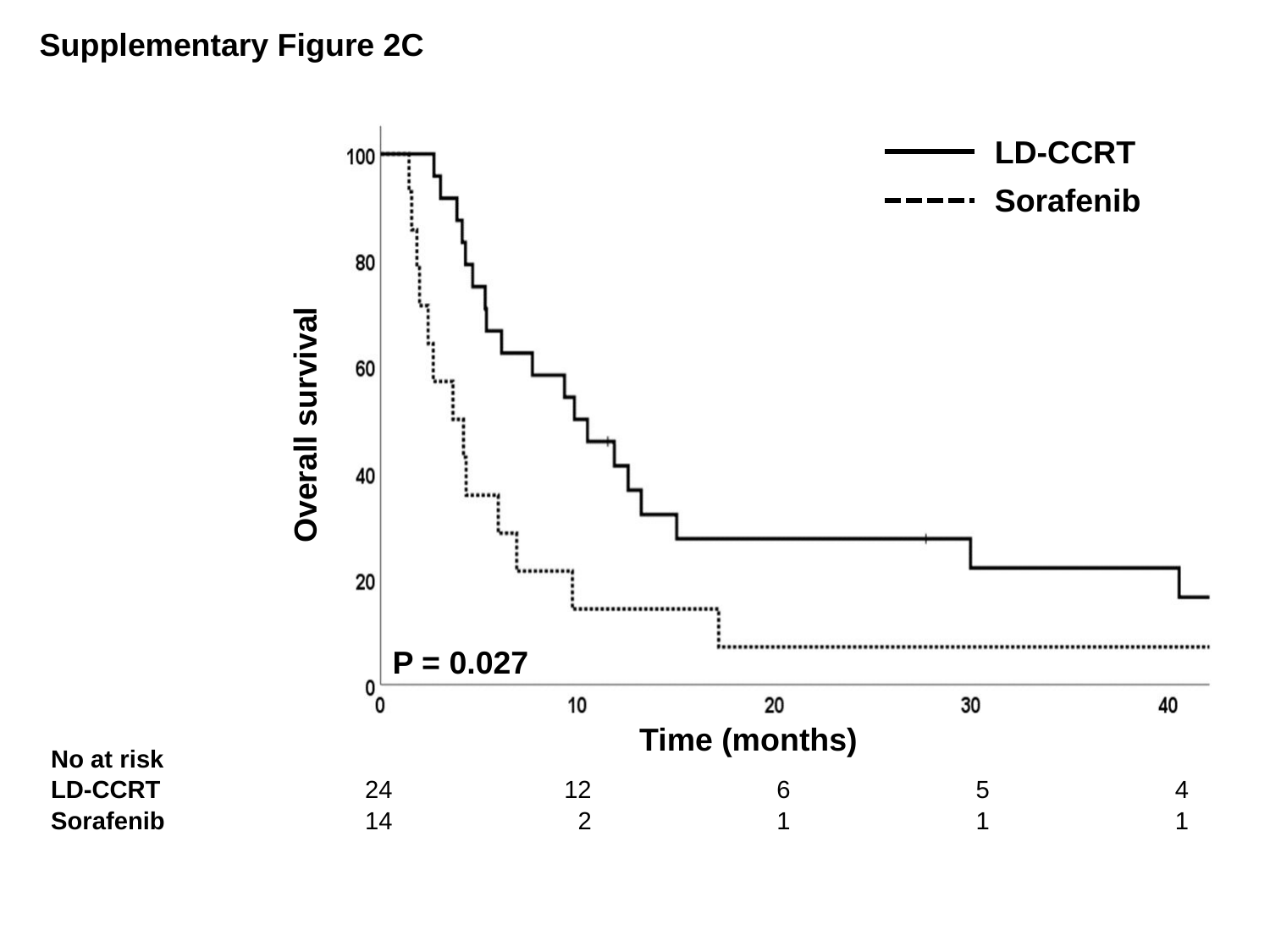

Supplementary Figure 2C
LD-CCRT
Sorafenib
Overall survival
P = 0.027
Time (months)
| No at risk | | | | | |
| --- | --- | --- | --- | --- | --- |
| LD-CCRT | 24 | 12 | 6 | 5 | 4 |
| Sorafenib | 14 | 2 | 1 | 1 | 1 |
